# Effect of Yoga versus Light Exercise to Improve Well-Being and Promote Healthy Aging among Older Adults in Central India: A Study Protocol for a Randomized Controlled Trial

**DOI:** 10.3390/geriatrics4040064

**Published:** 2019-11-16

**Authors:** Anita Choudhary, Ashish Pathak, Ponnaiah Manickam, Manju Purohit, Thomas Daniel Rajasekhar, Parag Dhoble, Ashish Sharma, Juhi Suliya, Dhanashree Apsingekar, Vandana Patil, Ashish Jaiswal, Sudhir Gwarikar, Josefine Östh, Maria Jirwe, Vinod Kumar Diwan, Mats Hallgren, Vijay Mahadik, Vishal Diwan

**Affiliations:** 1 Department of Physiology, R D Gardi Medical College, Ujjain 456006, India; dranitats@gmail.com; 2 Department of Pediatrics, RD Gardi Medical College, Ujjain 456006, India; ashish.pathak@ki.se (A.P.); uctharc@sancharnet.in (V.M.); 3 Department of Women and Children’s Health, International Maternal and Child Health Unit, Uppsala University, SE-751 85 Uppsala, Sweden; 4 Department of Public Health Sciences, Karolinska Institutet, SE 171,76 Stockholm, Sweden; manju.purohit@ki.se (M.P.); josefine.osth@ki.se (J.Ö.); Maria.Jirwe@ki.se (M.J.); Vinod.Diwan@ki.se (V.K.D.);; 5 National Institute of Epidemiology, Indian Council of Medical Research, Chennai 600077, India; manickam@nie.gov.in (P.M.); danielrosy@gmail.com (T.D.R.); 6 Department of Pathology, R D Gardi Medical College, Ujjain 456006, India; 7 Department of Psychiatry, R D Gardi Medical College, Ujjain 456006, India; dr_paragdhoble@yahoo.com; 8 Department of Medicine, R D Gardi Medical College, Ujjain 456006, India; ashishricha2001@yahoo.co.in (A.S.); rdgmc_ujn@sancharnet.in (S.G.); 9 Indian Institute of Public Health Gandhinagar, Gujarat 382042, India; drjuhee.saims@gmail.com (J.S.); dhanashree26a@gmail.com (D.A.); 10 R D Gardi College of Physiotherapy, Ujjain 456006, India; 11 Department for Health Promoting Science, Sophiahemmet University, SE 114 86 Stockholm, Sweden; 12 Department of Public Health and Environment, R.D. Gardi Medical College, Ujjain 456006, India; 13 International Centre for Health Research, Ujjain Charitable Trust Hospital and Research Centre, Ujjain 456006, India

**Keywords:** yoga, India, well-being, aging, RCT, physical activity, cognition, light exercise

## Abstract

Background: Aging is a natural process associated with many functional and structural changes. These changes may include impaired self-regulation, changes in tissues and organs. Aging also affects mood, physical status and social activity. There are adverse changes in cognitive behavior, perceived sensation and thinking processes. Regular physical activity can alleviate many health problems; yet, many older adults are inactive. Yoga is one of the scientific and popular lifestyle practice considered as the integration of mind, body and soul. Results of previous studies reported positive effects of yoga on multiple health outcomes in elderly. However, there is scarcity of scientific information where yoga’s effect is examined on over well-being and on multiple health outcomes simultaneously in elderly. This protocol describes methods for a 12-week yoga-based intervention exploring the effects of yoga on well-being in physically inactive elderly living in community. Methods and analysis: This two group parallel single blind randomized controlled trial that will be conducted at a designated facility of R.D. Gardi Medical College, Ujjain, Madhya Pradesh, Central India. A 12-week 60-min yoga intervention three times weekly is designed. Comparison group participants will undergo a 60-min program comprising light exercise focusing on conventional stretching to improve mobility. After screening, 144 participants aged 60–80 years will be recruited. The primary outcome is subjective well-being. Secondary outcomes include mobility, fall risk, cognition, anxiety and depression, mood and stress, sleep quality, pain, physical activity/sedentary behavior and cardio-metabolic risk factors. Assessments will be conducted at baseline (0 week), after the intervention (12+1 week) and at follow-up (36+1 week). Intention-to-treat analyses with mixed linear modeling will be applied. Discussion: Through this trial, we aim to determine whether elderly people in the intervention group practicing yoga show more favorable primary (well-being) and secondary outcomes than those in the light exercise focusing on conventional stretching group. We assume that yoga may be practiced to maintain health, reduce particular symptoms commonly associated with skeletal pain, assist in pain relief and enhance well-being. We anticipate that practicing yoga will improve well-being and mental health and may lead to significant improvement in depression, pain and sleep quality.Ethics and dissemination: This study is approved by the Institutional Ethics Committee of R.D. Gardi Medical College, Ujjain, IEC Ref No. 09/2018. All participants would be provided with written and verbal information about the purpose of the project and would be free to withdraw from the study at any time. Refusal to participate in the study would not have any negative consequences. Confidentiality of the information of each participant would be ensured. Knowledge obtained would be disseminated to stakeholders through workshops, meetings and relevant scientific conferences.Trial Registration: The trial is prospectively registered with the Indian Council of Medical Research Trial Registry CTRI/2018/07/015051.

## 1. Introduction

Aging is a natural process associated with many functional and structural changes [1,2]. These changes may include impaired self-regulation, changes in tissues and organs. Aging also effects mood, physical status and social activity, adverse changes in cognitive behavior, changes in perceived sensation and thinking processes [3]. The world’s population is aging, and more people are living longer. It is estimated that the proportion of the individual over 60 years will almost double from 12% to 22% between 2015 and 2050 [4] thus also increasing in prevalence of non communicable disease and non-fatal disabilities and in ultimately increases the dependency.

Physical activity (PA) is a protective factor for non communicable diseases such as cardiovascular disease, stroke, diabetes and some types of cancer [2]. PA is also associated with improved mental health [3], delay in the onset of dementia [5] and improved quality of life and wellbeing [5,6]. The health benefits of PA are well documented with higher levels and greater frequency of PA being associated with reduced risk and improved health in a number of key areas [7].

It is widely documented that regular physical activity in elderly can help in delaying and preventing a wide range of health problems including cardiovascular disease, stroke, diabetes and some types of cancer [6,7,8]. Regular physical activity can further show improvements in conditions such as pain, mobility, mood disorders, mental health, sleep quality, depression and anxiety [9,10]. Inspite of these known benefits, studies have documented that with the increase in age, the level of physical activity is decreasing [11]. The decrease in physical activity can have major negative consequences on the non- communicable diseases [7], which is to continue to be the one of the leading risk factor for mortality [12]. It is important to focus on interventions that improve healthy aging, reduces disability onset outcomes and enhances life quality.

The evidence from recent studies has identified the elderly’s interest in different type of physical activity that are simple, easy and help them in developing well-being and internal satisfaction than focused on fitness and appearances [13,14,15,16,17]. Well-being, which is consisted of physical, psychological and social components [18], and high well-being are associated with low prevalence of non communicable diseases, healthy aging and longevity [19,20]. 

Yoga, is one of the scientific and popular lifestyle practices considered as the integration of mind, body and soul [21]. Yoga has been proven to be extremely beneficial for physical, mental and social health since ancient times, and it is gaining popularity worldwide very rapidly [21,22].

Several studies conducted across the globe have demonstrated the positive effects of yoga on the number of health outcomes that concerns elderly such as improvements in depression and anxiety [13,23,24,25,26,27], mood and stress [25,28], pain reduction [29,30], enhanced sleep quality [13,25,26,30,31], balance resulting in fall prevention [23,27,32,33] cognition [31,34] and cardio metabolic health [17,28,30,35]. Studies have also reported improvement in the subjective well-being [36,37]. In elderly, yoga based intervention helps in maintaining breathing and heart rate, decreasing blood pressure, lowering cortisol levels and helps in increasing blood flow, which further helps in restoring autonomic regulatory reflex mechanisms associated with stress [30].Yoga appears to be one of the viable interventions to reduce inflammation in different chromic conditions [38]. Studies also reported the positive effect of yoga on inflammatory markers such as cytokines interleukin-6 (IL-6) and tumor necrosis factor (TNF) and the acute-phase protein C-reactive protein (CRP) [38,39].With few exceptions [31,40,41,42], the current evidence is limited primarily to ‘feasibility’, ‘exploratory’ and ‘pilot’ studies; many of which have been conducted in residential care homes. Moreover, most studies of yoga and cognition/mood have not included older adults. What is now required are rigorously designed and adequately powered community-based trials that include long-term assessments of multiple and diverse health outcomes.

### 1.1. Rationale

Injury related hospitalizations are on the increase in the elderly population. Fall injuries are more prevalent in elderly, which accounts for 10–15% of all emergencies. Falls are also the leading cause of injury-related death in the elderly as well. Healthy is considered as the process of developing and maintaining the functional ability that enables wellbeing. Though normal exercise is recommended to prevent several non communicable diseases [43], however due to its focus on flexibility and balance yoga can help more in reducing the risk of fall-related injuries [16,42]. Further with its multidimensional and holistic focus on the physical, mental and spiritual approach, yoga may be more suitable and manageable than normal exercise for elderly [44]. Further yoga emphasized on breath regulation, mindfulness and maintenance of postures, which is not a part of normal exercises [45]. Studies previously conducted, compared the effect of yoga on non-active controls [46]. No studies have been reported where effects of yoga on subjective well-being is compared to light exercise designed to improved mobility among elderly in a community setting in developing countries.

The present study protocol proposes the methodology of an RCT that aims to improve the health and well-being of elderly between 60–80 years through yoga-based interventions. We will explore the effects of participation in a yoga-based program on subjective well-being (primary study outcome) and healthy aging in older adults; the primary outcome will be measured using the life satisfaction index, version Z (LSI-Z) and satisfaction with life scale (SWLS). We will assess whether the practice of yoga results more favorable health outcomes than the practice of light mobility exercises. The potential public health implication of this study will be immense if the study results able to demonstrate that yoga has a positive effect on elderly’s well-being, mobility and cognition. This may further help in the prevention and treatment of age related diseases and conditions and development of future preventive interventions for the elderly in community setup. We are also anticipating that the project will also empower participants to initiate and continue yoga-based activities beyond the intervention. We are anticipating that the benefits of the intervention will be felt by the participants, their families and society in the form of (anticipated) lower health costs.

### 1.2. Study Objective

This randomized controlled trial will (1) examine the effects of a 12-week yoga based intervention focused mainly on physical postures and movements on subjective well-being among healthy but physically inactive elderly Indian aged 60–80 years and(2) study the effect of a yoga based program on multiple secondary health outcomes affecting elderly. The comparison group with equivalent duration will be light exercise focusing on conventional stretching and mobility.

### 1.3. Specific Objectives

The specific objectives of this study are to study the effect of a 12-week yoga-based intervention compared to light exercise focusing on conventional stretching intervention in elderly Indian aged 60–80 years on:

- Subjective well-being;
- Prevalent multiple health outcomes such as poor sleep quality, pain, depression anxiety, mood and stress;
- Balance and fear of falling and physical activity levels;
- Cognitive function;
- Cardio-metabolic measures such as blood pressure, blood glucose and blood lipids and on cortisol and other inflammatory markers.

### 1.4. Hypotheses

We hypothesize that compared with light exercise intervention focusing on conventional stretching and mobility; a yoga-based intervention of same duration would result in moderate improvements (20%) in self-reported subjective well-being and similar magnitude improvements on all other secondary outcomes (pain, mobility, sleep, cognition, mood, stress and cardio metabolic and inflammatory markers).

### 1.5. Trial Design

The trial will be conducted according to Consolidated Standards of Reporting Trials (CONSORT) guidelines, and the protocol is drafted in accordance with Standard Protocol Items: Recommendations for Interventional Trials (SPIRIT) guidelines.

**Figure 1:**
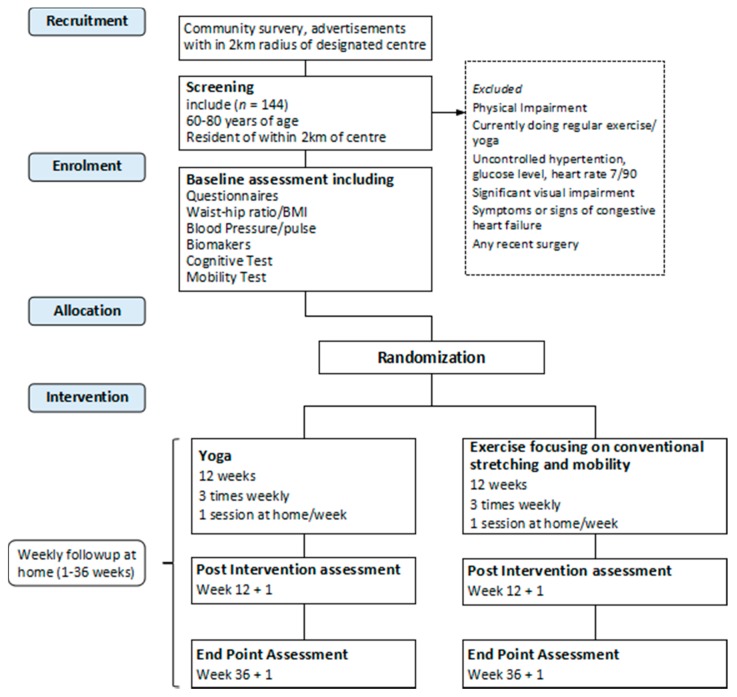
Design and participant flow chart according to SPIRIT 2013 guidelines (Standard Protocol Items: Recommendations for Interventional Trials).

The study is designed as a two-arm, exploratory, single-blinded RCT. Participants will be randomize into two groups receiving yoga and light exercise focusing on conventional stretching and mobility. Assessment will be performed at baseline (0 week), after the intervention (after 12+1 week) and at follow-up (after 36+1 week; Figure 1 and Table 1).

## 2. Methods

### 2.1. Study Setting

The intervention will be conducted at the designated facility of R.D. Gardi Medical College, Ujjain, Madhya Pradesh, India. Assessments of primary and secondary outcomes will be conducted at R.D. Gardi Medical College and its associated hospitals.

### 2.2. Inclusion Criteria

Elderly aged between 60 and 80 years who will be willing to participate and who resided in a locality within 2 km from the designated intervention facility will be included. This may also include elderly living in home with family or independently.

### 2.3. Exclusion Criteria

- Individuals currently enrolled in any other research study;
- Individuals advised by doctors to not perform exercises;
- Individuals involved in current or recent (last two months) practice of yoga or those with regular (three times per week or more) participation in other planned exercises, such as aerobics or strength training;
- Individuals with health or mobility problems that would interfere with yoga training such as paralysis or inability to sit, stand, walk and severe pain;
- Individuals having evidence of liver or kidney dysfunction, significant lung diseases, symptoms or signs of congestive heart failure, ischemic heart disease or significant valvar disease and significant visual impairment;
- Individuals having a resting heart rate >100 beats/min, uncontrolled diabetes mellitus with blood glucose measuring greater than or equal to 400 mg/dL and blood pressure >160 mmHg systolic, >100 mm Hg diastolic;
- Individuals having major surgery in the last year or planning to undergo future surgeries.

### 2.4. Interventions

#### 2.4.1. Yoga

Elderly who will be randomized to the yoga group will perform the yoga-based intervention three times per week during 12 weeks at a designated facility. The participants will also be encouraged to complete at least one additional yoga session of the same duration at home per week. Yoga will be conducted for 60 min as a classroom session and will accommodate approximately 15–20 participants. Two qualified yoga instructors (male and female) will teach in these classes. The classes will be design to be safe and suitable for elderly and will focused on improving flexibility, balance and strength. Both males and females will be in the same class. The duration of the intervention will be similar to other yoga-based programs reported in literature suitable for elderly [22,47].

The yoga intervention will be standard throughout the study to avoid the risk of intervention variability. Our proposed yoga intervention is based on Hatha Yoga that included physical postures (asanas), breathing exercises and moderate meditation [48]. The following yoga poses will be performed in this order each time: Crocodile pose (Makrasana), Palm tree pose (Tadasana), Hands to feet pose (Hastpadasana), Chair pose (Utkatasana), Twisted pose (Vakrasana), Angle pose (Konasana), Lotus Pose (Padmasana), Yogmudra, Seated Forward Bend Pose (Paschimotanasana), Cobra Pose (Bhujangasana), Locust Pose (Shalabhasana), Wind Relieving Pose (Pawanmuktasana), Stick Pose (Yastikasana), Corpse Pose (Shavasana), Crossed Legs Pose (Sukhasana) and OM Chanting. The description of these poses are described in detail elsewhere [48].

A thirty minute session will be organized by yoga instructors after each session during the first week of the class to give detail instructions regarding the home session. Thereafter every class the yoga instructors will remind participants about the home session. Additionally, a booklet will be given to all participants of yoga group where all instructions, precautions and description of poses and asanas will be described with pictures.

#### 2.4.2. Light Exercise Focusing on Conventional Stretching

In this study we will have active light exercise comparison group to increase the internal validity and adherence. Our comparison group will consist of light exercise focusing on conventional stretching designed to improve mobility. This comparison group will be a genuine light exercise training condition with no strength-training, yoga, meditation or breathing exercises included. The rationale for including such a comparison group, where participants underwent an exercise program at the same duration, frequency and location as the yoga intervention group, is to reduce the likelihood of the Hawthorne effect. This would also make participants happy—but nothing that would give the benefits of real yoga. The light exercise program will include standing positions exercises such as movements of fingers, wrist, elbows, shoulders and legs. The sitting position exercise will include joints movements of the toe, ankle and knee. Lie down exercises will include leg rotation, knee bending and cycling. The comparison group participants will copy the instructor’s movements to ensure that they are not exhausting and strenuous. Engagement of comparison group participants will be done at a similar location and will have the same frequency and duration of training as the yoga group (i.e., 60 min of session thrice a week during 12 weeks at the designated facility and at least one additional session of the same duration at home per week). The sessions will be conducted as a class room session at the same location as the yoga intervention under the supervision of trained instructors (male and female). Each classroom will accommodate approximately 15–20 participants. Both males and females will be in the same class.

A thirty minute session will be organized by trained exercise instructors after each session during the first week of the class to give detail instructions regarding the home session. Thereafter every class the instructors will remind participants for home session. Additionally, a booklet will be given to all participants of the comparison group where all instructions, precautions and descriptions of exercises will be described with pictures.

### 2.5. Follow-Up Visits

**Figure 2:**
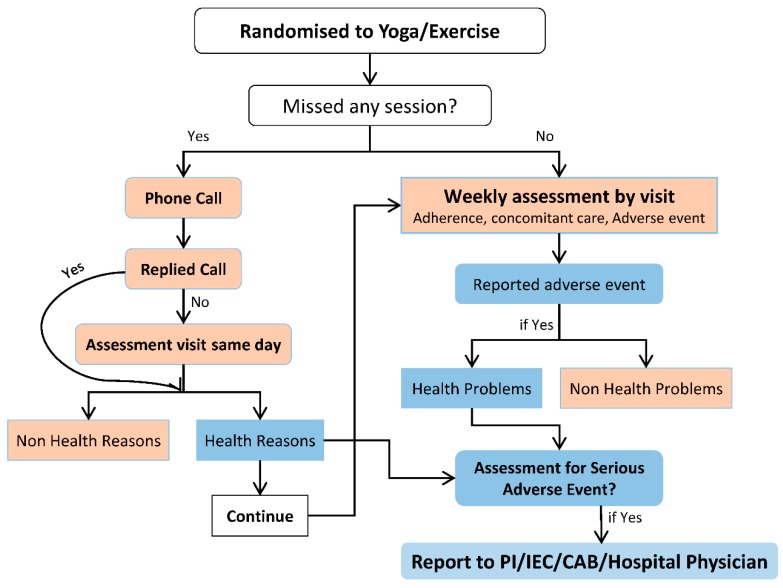
Follow up flowchart.

All the participants will be weekly followed up from the first week to the 36th week. Participants who will miss any session will be telephonically contacted on the same day by research assistants to enquire about the reason for nonattendance. Participants who will not attend a session due to health reasons will be visited by a research assistant on the same day to document the events. Additionally, all participants will be visited physically by research assistants at home once a week to document adherence (home exercise/yoga), any self-modification in intervention, health-seeking behavior and to record any adverse events in a pre-designed follow-up form (Figure 2).

### 2.6. Strategies to Maintain Adherence

The literature has shown that adherence to physical activity training is low, and this trend is more common in the elderly age group [49]. To minimize participant dropout, one thirty minute face to face session will be organized before the start of the project, in which the project team will communicate with prospective study volunteers. The project team will inform all the components of the study and will address all queries and concerns of participants. All sessions will be conducted by qualified instructors (male and female) of age more than 45 years so that participants would feel less hesitant in following the classes. The yoga and aerobic exercise classes will be especially designed for elderly people. Participants will be provided access to free emergency medical care at all times. All assessments and samplings will be done free of charge at all the three assessment points. The study participants will be recruited within 2 km radius of the facility to minimize the travel time. A pickup and drop facility will also be arranged by the project team if demanded specifically. Daily attendance of participation will be maintained. Weekly follow-up visits will be conducted by research assistants to participants’ homes to document about home session adherence, any changes/modification in yoga exercise intervention at home and documentation of adverse events. Both the classes will be conducted free of charges.

### 2.7. Outcomes Variables

#### 2.7.1. Primary

Well-being will be measured subjectively using the 13 items life satisfaction index, version Z (LSI-Z) [50,51]. Five core components of life satisfaction are measured in the life satisfaction index, namely zest, resolution and fortitude, congruence between desired and achieved goals, positive self-concept and mood tone [52]. Subjective well-being will also be assessed by using a five-item satisfaction with life scale (SWLS) that measures global judgments of one’s life satisfaction [53]. On this scale, the participants show how much they agreed with or disagreed with each item on a scale of 7.

#### 2.7.2. Secondary

- Physical activity and sedentary behavior will be assessed using the short form of the International Physical Activity Questionnaire (IPAQ) [54,55] and the five-item Simple Physical Activity Questionnaire (SIMPAQ) [56].
- The Berg balance scale, a 14-item objective measure that assesses static balance and fall risks in adults [57,58,59] and modified falls efficacy scale (MFES) [60,61], a 14-item activity questionnaire, which assesses the fear of falling among older adults in various indoor and outdoor activity, will be used to measure mobility and falls.
- Self-reported depression will be measured using the geriatric depression scale (GDS), which is a 30-item screening instrument for older adults [62,63].
- Self-reported anxiety will be assessed using the geriatric anxiety inventory (GAI) scale.
- Mood states and psychological distress will be assessed using the profile of mood state questionnaire (POMS) [64].
- The perceived stress scale (PSS) will be used to assess pass month subjective stress [65].
- The brief pain inventory (BPI) short form will be used to measure pain and its severity [66,67].
- Self-reported sleep quality will be measured using the insomnia severity index (ISI), which is a seven-item self-rated questionnaire [68,69].
- Cognition will be measured using the mini-mental state examination (MMSE), which is an 11-item questionnaire that tests five areas of cognitive function [70,71].
- Body mass index (BMI), resting heart rate, blood pressure, waist–hip ratio (WHR), blood glucose, blood lipids and cortisol will be collected to assess cardio metabolic risk factors. Interleukin-6, and tumor necrosis factor-alpha will also be also examined.

#### 2.7.3. Sample Size

The calculation of sample size for this study is based on the effect size reported in a six month long RCT where results of Iyenger yoga on elderly was compared with walking [42]. Based on this study we anticipate that the mean difference (effect size) of around 0.2 on our primary outcome that is well-being in yoga intervention group compared to exercise group. With 80% power, two-tailed significance for the primary between-group comparisons and 1:1 allocation, a total sample of 120 participants is estimated. Assuming a 6-month dropout rate of 20% [72], the total number of participants required at baseline will be 144.

#### 2.7.4. Recruitment

Recruitment of participants will be conducted using multiple methods of placing advertisements in local newspapers, contacting local pensioner clubs and old age groups and conducting door-to-door surveys in the two-kilometer vicinity of the designated intervention facility. Prospective participants will be provided detailed information of the study. Interested participants will be requested to visit the designated facility at the designated time for eligibility screening. Additional detailed information on the study will be provided verbally and in writing in the local language to all prospective participants prior to eligibility screening. During the course of study we aim to screen at least 600 individuals for eligibility. We anticipated that every fourth volunteer initially selected will be interested and eligible to participate in the study. At each phase we will enroll 15–20 participants, resulting in a total of 144 participants. Staggering the recruitment in this way will make the process more manageable and logistically feasible. The recruitment drive will be done at least 6–8 times until the required number is achieved. New participants will be added to the existing group as per the availability in the class and space vacated by previously admitted participants. Eligibility screening will be performed in week −1 at the designated medical facility of R.D. Gardi Medical College, Ujjain by a team comprising of physician, physiotherapist, psychologist and supported by research assistants trained in social work and public health. The composition of the team that would measure the study outcomes will be consistent throughout the project.

Consent to participate: All the participants will be individually introduced to the study in a face-to-face session with project coordinator and instructors. Participants will be provided with verbal and written information about the purpose of the study, confidentiality and rights to withdraw at any time. Written informed consent will be sought from all the study participants. All data will be analyzed and reported at the aggregate level only; no individual patient data will be released.

#### 2.7.5. Randomization and Blinding

The randomization sequence by gender will be generated by an external statistician who is not directly involved in the implementation of the intervention. Each allocation sequence will be transferred into separate sealed envelopes by an assistant nominated by the statistician, which will not be part of the study. Immediately after baseline assessments, the allocation sequence in a sealed envelope will be opened by the principal investigator or project coordinator together with eligible participants. Participants will be informed whether they are allocated to the yoga or light exercise group. At baseline, participants will be requested to not discuss their treatment allocation with the research team responsible for conducting follow-up assessments where a different set of research team blinded to the participant’s allotted group will conduct these assessments. The allotted group will be kept confidential and will be un-blinded only if participants withdraws or due to adverse events. The instructors will also be informed about the allotted group for proper management of yoga/exercise classes.

### 2.8. Data Collection

#### 2.8.1. Time Points

Primary and secondary outcomes will be measured at baseline (0 week), post-intervention (12+1 week) and at end point (36+1 week). The assessments will include basic demographic-socio-economic information, anthropometrics measurements, primary and secondary outcome measurements and biological sampling (see Figure 1 and Table 1).

#### 2.8.2. Baseline Assessment 

After participants’ inclusion and after their signed informed consent is obtained, baseline assessments will be conducted prior to the randomization by the trained project assistants Assessments of demographic-socio-economic characteristics, anthropometric measurements and primary and secondary outcomes (see the outcome variables section) will be conducted. It is anticipated that a total of 120 min–140 min will be required to complete all baseline assessments. If participants are not willing to give information at one sitting, the next possible dates will be discussed and assessments will be made as per the participant’s convenience.

#### 2.8.3. Post Intervention and End Point Assessments 

Post-intervention assessments and end point assessments will be conducted at 12+1 and 36+1 weeks, respectively. All assessments and blood analysis will be similar to baseline assessments. It is anticipated that a total of 100 min–120 min will be required to complete all post intervention and end point assessments.

#### 2.8.4. Biological Sampling 

Before all three assessments ten mL of blood samples will collected by phlebotomists one or two days prior to the administration of questionnaires as per the convenience of participants to measure fasting glucose, complete blood count, lipid profile, cortisol, CRP and biomarkers in the morning hours (empty stomach) between 6.30 a.m.–7.30 a.m. by visiting participants home. A 5 mL post prandial blood sample will be collected after two hours of eating a meal between 1:00 p.m.–3:00 p.m. by visiting the participant’s home. The sample will be collected in EDTA, plain, fluoride vial and will be immediately transferred under a cold chain condition to the central research laboratory of R.D. Gardi Medical College for further analysis. Three mL of sample will be stored in −20 degrees for further analysis of biomarkers. Standard methods will be used for the analysis of samples.

### 2.9. Follow-Up Visits

All the participants will be weekly followed up from the first week to the 36th week. Participants who will miss any session will be telephonically contacted on the same day by research assistants to enquire about the reason for nonattendance. Participants who will not attend a session due to health reasons will be visited by research assistants on the same day to document the events. Additionally, all participants will be visited physically by research assistants at home once a week to document adherence (home exercise/yoga), any self-modification in intervention, health-seeking behavior and to record any adverse events in pre-designed follow-up form (Figure 2). 

### 2.10. Data Management

The principal investigator and study coordinator will be responsible for data management, record keeping, safety and timely backups. Data will be collected in a confidential participants case report form (CRF), which will be later transferred into electronic form (Microsoft Excel). Each participant will be given a unique participation information number (PIN) to de-identify the study participant and to ensure patient anonymity and data confidentiality. The entered data will be transferred to appropriate statistical software for further analysis.

### 2.11. Community Advisory Board

An independent community advisory board (CAB) will be formed to monitor the interventions. The board will comprise of community members including a trained health professional, physical exercise trainer and retired college professor who will hold discussions with the study participants about the process, interventions and other related issues. They will also provide participants’ feedback to the project team. The project team will inform the CAB about any adverse events and the action taken.

## 3. Statistical Analyses

We will use appropriate parametric (*t*-test and Analysis of variance (ANOVA) and non-parametric (Mann–Whitney U and Kreskas Wallis test) tests to compare the socio-demographic-economic and other base-line characteristics of the two groups. Intention-to-treat (ITT) analyses to examine the effects of the intervention that will be explored using mixed linear modeling, in which group × time interactions will be reported with regression estimates, confidence intervals and effect sizes. Sensitivity analyses will also be performed. The moderating effects of gender and age and the interaction effects of education on cognitive outcomes will be also be examined. A similar plan will be followed for the analysis of secondary outcomes.

### 3.1. Assessment of Adverse Events

In the proposed study, the potential risks of adverse events are minimal. Most frequently anticipated adverse events are muscle strains, soreness, knee pain, Achilles tendon, back spasms, neck pain, vertigo, migraine and reoccurrence of prior lower back pain or shoulder problems [73].All sessions will be conducted by qualified yoga instructors at a health center or near a health center in Ujjain. Participants will be provided access to emergency medical care at all times. The interventions will be specially designed for elderly. A team of physicians will screen all potential participants and will exclude those with health problems that may hinder participation..All adverse events will be recorded, and followed up by research assistants. Adverse events will be formally assessed during the weekly home visits of study participants where participants will be asked to inform project team of any health related events they encountered in past week. Adverse events will also be communicated to the team of physicians for assessments and medical management.

### 3.2. Ethics Approvals

The study will be conducted in accordance with the protocol, Good Clinical Practice as well as the requirement from the Institution’s Ethical Committee. The study is approved by the institutional ethics committee of R.D. Gardi Medical College, Ujjain (IEC Ref. No.-09/2018). The study is also registered in CTRI (Clinical Trials Registry India) Indian Council of Medical Research (CTRI/2018/07/015051).

## 4. Discussion

With this study we aimed to explore whether the effects of yoga-based intervention differ from those of light exercises in community elderly individuals in an Indian setting. Eligible participants will be individually randomized (after completing baseline assessment) to either 12-weeks of intervention of either a supervised yoga-based program or light exercises. Participants will be allocated to permuted blocks to achieve a gender balance in the two groups. The randomization procedure will be performed externally using a random number generator.

A systematic review concluded that studies of yoga lack clinical evidence [74]. We anticipated our trial would contribute to the current evidence on the effects of yoga interventions on various components of physical and mental well-being. Through this trial, we assumed that comparison of yoga and light exercise focusing on conventional stretching to improve mobility groups will document changes in the outcomes measured such as well-being, mobility, depression, pain, sleep quality and physical activity. We will minimize the barriers to learning and practice among participants and instructors by ensuring a small age gap between participants and instructors; hence, participants will feel less hesitant in following the classes. This may prove to be a comfortable and suitable setting compared to conventional yoga/fitness classes.

Previous studies of effectiveness of yoga have investigated some of the outcomes that will be measured in our study [14,75], however, measuring a range of relevant and meaningful outcomes (as primary and secondary outcomes) simultaneously may provide credible evidence to assess if yoga is more beneficial than light exercises. For elderly, yoga may provide a broad range of healthcare benefits for the mind and body. Yoga may be practiced to maintain health, reduce particular symptoms commonly associated with skeletal pain, and assist in pain relief, and enhance well-being [31,76,77]. We anticipate that practicing yoga may improve subjective well-being and mental health and may result in significant improvement in depression, pain and sleep quality. Furthermore, regular yoga practice can increase mindfulness of the body state and can restore the mind–body balance among elderly people [48,78,79]. The intervention may encourage participants to develop further interest in continuing the physical activity even after the completion of the study.

The present understanding came from mainly studies conducted in residential care homes [80]. Most of other studies did not included elderly. There is a requirement of well-designed community-based follow studies to assess long term changes and outcomes. We feel that with yoga being a practical, easy and feasible exercise that it can help in emotional and physical betterment and ultimately wellbeing in an elderly group. This project aimed to encourage participants to initiate and continue a yoga-based activity after study completion. We anticipate significant improvements in several health domains. The intervention would benefit participants, their families and society in terms of (anticipated) lower health costs. Moreover, the study results may be of broader use in other settings. We would use the standardized measurements and blinded follow-up assessments, to reduce the assessment bias.

However, there may be some possible limitations in this study. This study concerns with the overestimation of intensity/duration of physical activity assessed through subjective measurement, due to social desirability of responses i.e., there is a possibility that contact with other participants/trainers/research assistants may increase wellbeing in studied population. Generalizability of study will be limited to similar settings and conditions. We will recruit only those individuals that will show interest towards devoting time in physical activities.

## Figures and Tables

**Table 1:** Schedule of enrolment, interventions and assessments, according to SPIRIT 2013 guidelines. BMI, body mass index; SPIRIT, Standard Protocol Items: Recommendations for Interventional Trials; WHR, waist-hip ratio, TNF-α, Tumor necrosis factor alpha; IL-6, Interleukin6; CRP, C-Reactive Protein.

Study Period						
**Time Point**		**Enrolment**	**Baseline**	**Intervention**	**Post Intervention**	**Endpoint**
		week −1	week 0	week 1–12	week 12+1	week 36+1
**Eligibility Screen**		✓				
Informed Consent		✓				
Allocation		✓				
**Intervention**						
Yoga				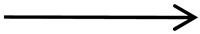		
Light Aerobic Exercise				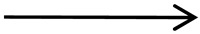		
**Study Outcomes**	**Methods for Assessment**					
Demographic	Socio-economic questionnaire		✓		✓	✓
Well-being	Life Satisfaction Index Z Satisfaction with Life Scale		✓		✓	✓
Mobility/fall risk	Modified Falls Efficacy Scale Berg Balance Scale		✓		✓	✓
Pain	Brief Pain Inventory		✓		✓	✓
Mood	Profile of Mood Status		✓		✓	✓
Stress	Perceived Stress Scale		✓		✓	✓
Anxiety	Geriatric Anxiety Inventory		✓		✓	✓
Depression	Geriatric Depression Scale		✓		✓	✓
Physical Activity	International Physical Activity Questionnaire		✓		✓	✓
Sedentary behavior	Simple Physical Activity Questionnaire		✓		✓	✓
Sleep quality	Insomnia Severity Index		✓		✓	✓
Cognition	Mini Mental State Examination		✓		✓	✓
Cardio Metabolic Risk	Blood pressure, resting heart rate, BMI, WHR		✓		✓	✓
Blood tests	Complete blood count, Blood glucose, Blood lipids, IL-6, TNF-α, CRP, Cortisol		✓		✓	✓
Followup	Followup visit to participant’s home to check adherence/modification/adverse events			Weekly home visit from week 1 to week 36		
Feedback	Feedback and participants satisfaction					✓
